# Amperometric systems for the determination of oxidase enzyme dependent reactions by continuous flow and flow injection analysis

**DOI:** 10.1155/S1463924680000570

**Published:** 1980

**Authors:** E. L. Gulberg, A. S. Attiyat, G. D. Christian

**Affiliations:** 1 Department of Chemistry University of Washington Seattle WA 98195 USA; 2 Department of Chemistry Yarmouk University Irbid Jordan


### Amperometric systems for the determination of oxidase enzyme dependent reactions by continuous flow and flow injection analysis

E. L. Gulberg
*Department of Chemistry, University of Washington, Seattle, WA 98195, USA.*

A. S. Attiyat
*Department of Chemistry, Yarmouk University, Irbid, Jordan.*

G. D. Christian
*Department of Chemistry, University of Washington, Seattle, WA 98195, USA.*

### Introduction

There are two primary types of flow analysis. One is continuous flow analysis (CFA). In Skegg's classic CFA system the attainment of a steady state and air segmentation, which serves as a regulator to the flow [1] as well as a physical barrier to dispersion, are essential features [1,2,3]. The signals, commonly in the form of a steady state plateau, are usually dependent on the sample dispersion and mixing [3].

A second type of flow analysis is flow injection analysis (FIA), described first by Bergmeyer and Hagen [4], and later by Ruzicka and Hansen [5] and Stewart and co-workers [6]. In this system the sample is introduced as a plug into a flowing stream via a valve or syringe, and mixing is accomplished by diffusion [2]. In contrast to CFA, the signal in FIA does not reach a steady state plateau, but gives sharp peaks [2]. FIA has the advantage of a sampling rate commonly over 120 per hour, and as high as 300 per hour [7] or more [8,9]. The several applications of flow injection analysis have been reviewed by Betteridge [2].

While optical detection methods have been very successful in continuous flow and flow injection analysis, many electrochemical techniques have also been used for detection [2,10]. Voltammetric measurements with tubular platinum electrodes have been used in flow systems [11]. Procedures have been described for the determinaton of lactate [12], triglycerides [13], and ethanol [14] using open tubular carbon electrodes by biamperometric monitoring of hexacyanoferrate [11] ion. Glucose and glucose oxidase [15,16] have been determined in serum, plasma, and whole blood by reacting with the $H_2O_2$ produced in the enzymatic reaction with iodide. The iodine produced is measured biamperometrically in static or stirred solutions.

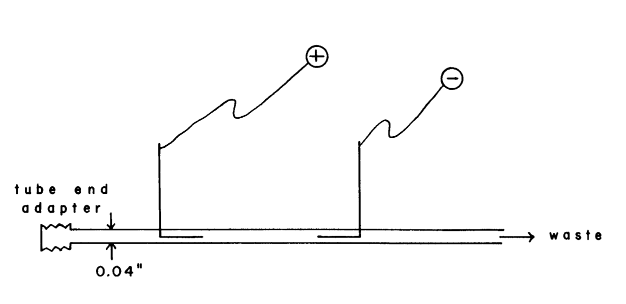

*Figure 1. Flow through biamperometric detection cell. Anode and cathode are thin (0.3 mm) platinum wires.*

In the present study several electrode systems were incorporated into a flow system to amperometrically or biamperometrically detect iodine in the presence of excess iodide. Both CFA and FIA systems were used to measure $H_2O_2$ and also ethanol using the reactions:

$$CH_3CH_2OH + O_2 \xrightarrow{\text{alc. oxidase}} CH_3CHO + H_2O_2 \quad (1)$$

$$H_2O_2 + 2I^- \xrightarrow{\text{Mo(V1)}} 2H_2O + I_2 \quad (2)$$

### Experimental

### Reagents

*Buffer:* Baker reagent grade sodium acetate and sodium phosphate were adjusted to pH values between 4 and 6, and 6 and 8, respectively, using concentrated HCl and 2MNaOH. TRIS (Sigma Chemical Co., St Louise, USA) was adjusted to pH values between 8 and 10 in the same manner. Tenth molar maleic acid (MC&B Manufacturing Chemists 2909 Highland Ave, Norwood, OH, USA) was used at pH 1 to 7.

*Potassium iodide:* Reagent grade KI (MC&B) was prepared in 0.1 M concentration and used in unbuffered aqueous solutions in the iodine determinations. In most of the other experiments 0.1 M KI was prepared in either 0.1 or 0.2 M acetate buffer (pH values between 4.7 and 5.0) containing $10^{-3}$ M ammonium heptamolybdate (Baker reagent grade). In the biamperometric determination of $H_2O_2$ using a platinum working electrode and a silver counter electrode, $10^{-3}$ M KI was used in 0.1 M acetate buffer, pH 5.0, containing$10^{-3}$M ammonium heptamolybdate and 0.2 M KCl.

*Hydrogen peroxide:* $H_2O_2$ standards were prepared fresh daily from nominally 3% $H_2O_2$ (MC&B).

*Ethanol:* Aqueous standards were prepared from absolute ethanol (U.S. Industrial Chem., New York, USA). All concentrations were prepared in volume/volume %.

*Alcohol oxidase* (from Candida Boidinii): Lot #77C-0314 was obtained from Sigma Chem. Co.

### Instrumentation

*Tubing:* All the tubing used was standard manifold pumping tubing from Gilson Medical Electronics and ranged in inner diameter from 0.02 to 0.1 inches.

*Pump:* A Gilson Minipulse 11 four channel persitaltic pump was used in all experiments.

*Flow-cell and electrodes:*

i) Two platinum wires approximately 0.002 in in diameter were inserted through the walls of a 0.04 in i.d. tygon tube and bent so that they protruded about 0.12 in down the centre of the tube. The two wires were separated by 0.31 in (Figure 1). Leakage through the walls of the tubing through which the wires were inserted did not occur.

ii) A single platinum wire as above was used with a saturated calomel electrode (SCE). The SCE reference was placed in a beaker into which the sample stream is fed directly.

iii) A platinum wire was cathodised for 30 minutes at 2mA in a $AuCl_4^-$ solution containing KCN. The gold plated wire was then inserted into the flowing stream as above with an SCE reference.

iv) A platinum wire working electrode was inserted into the tubing as above. A silver tube served as the counter electrode (Figure 2).

*Voltage source:* A Princeton Applied Research Polarographic Analyser (PAR 174) was used to provide a constant potential between the electrodes and to monitor the steady state current output. A Moseley X-Y recorder was used to record the signals and insure current levelling between standards and samples.

*Enzyme column:* Alcohol oxidase was immobilised by covalent attachment via glutaraldehyde on silanised glass beads and also on the inside walls of nylon tubing as previously described [17,18]. The glass bead column was approximately 8 in in length and 0.12 in in diameter. The silanised glass, packed into the column along with some larger, un-silanised beads, contained between 150 and 200 units of activity per gram, resulting in a column activity of approximately 10 units. The nylon type of column, 2 feet in length and 0.4 in in diameter, contained approximately 1 unit of activity.

### Procedure

*Iodine – CFA – twin platinum electrodes*

Aqueous standards of iodine (unbuffered) were prepared. A single 0.03 in i.d. inlet tube was run through the pump and connected directly to the flow cell. A blank of 0.1 M KI was followed by $I_2$ standards. With a potential of 200 mV poised between the electrodes, the biamperometric current due to the concurrent oxidation of iodide and reduction of iodine was recorded. A flow rate of between 0.3 and 0.8 ml per minute was used in this and in all following procedures.

*$H_2O_2$ – CFA – twin platinum electrodes*

Two separate 0.03 in i.d. inlet tubes were used to introduce the reagent (0.1 M KI and $10^{-3}$ M heptamolybdate in 0.1 M phosphate buffer, pH 7.8) and standard solution of $H_2O_2$ (the same buffer is used) into a mixing chamber. From the mixing chamber to the electrodes was a 35 cm length of tubing in which reaction 2 occurred. The pump speed was set so that the reaction time was 22 seconds. (A $10^{-5}$ M solution of $H_2O_2$ required 12 seconds for 90% oxidation of $I^-$ to $I_2$ at pH 5.0). As soon as the biamperometric current (at 200 mV) stabilised, a new $H_2O_2$ standard was introduced.

*Ethanol – CFA – twin platinum electrodes*

The flow system design for the determination of ethanol is shown in Figure 3. Ethanol standards in 0.05 M TRIS buffer at pH 8.2, were perfused through an immobilised alcohol oxidase enzyme column where reaction 1 took place. At the mixing chamber downstream from the enzyme column, the effluent from the column was mixed with a 0.2 M acetate buffer at pH 4.7, which contains 0.1 M KI and $10^{-3}$ M heptamolybdate. These reactants travelled along the reaction tube for about 25 seconds where reaction 2 occurred at a pH of about 5.0. The resulting $I_2$ was measured in the flow cell at an applied potential of 200 mV.

*$H_2O_2$ – FIA – twin platinum electrodes*

Three techniques were used to inject $H_2O_2$ standards into a reagent stream of 0.1 M KI and $10^{-3}$ M heptamolybdate in 0.1 M acetate buffer of pH 5.0. In one scheme, reagent and a blank of 0.1 M acetate buffer were pumped through separate 0.03 in i.d. tubes and mixed in a mixing chamber. Injections of $H_2O_2$ were made by transferring one inlet tube from the blank to a $H_2O_2$ standard in the same buffer. The sample was allowed to perfuse for 10 seconds (approximately 50 $\mu$l) at which time the tube was transferred back to the buffer blank.

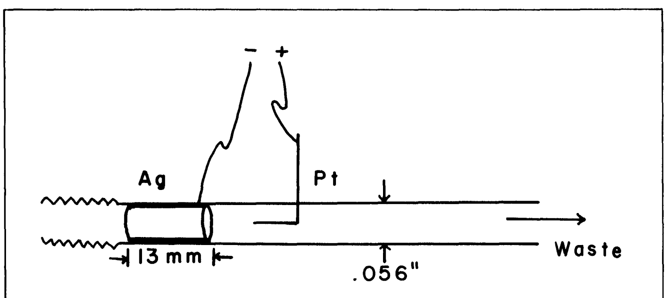

*Figure 2. Flow through detection cell with platinum wire anode and silver tube cathode.*

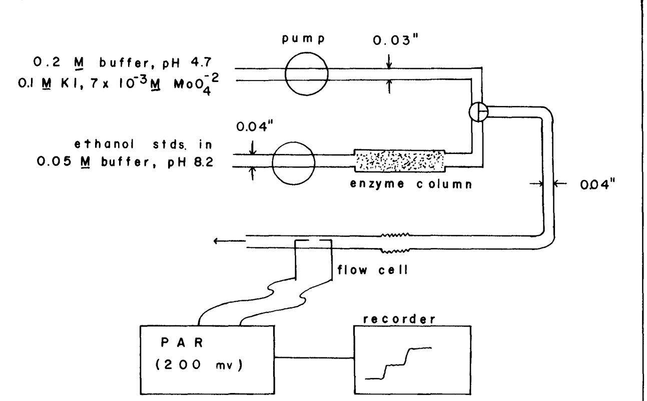

*Figure 3. Flow system for ethanol determination using immobilised alcohol oxidase (CFA). Effluent from mixing chamber to flow cell has a pH of 5.0.*

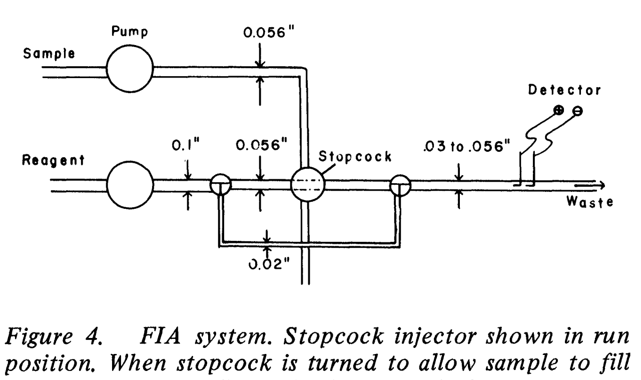

*Figure 4. FIA system. Stopcock injector shown in run position. When stopcock is turned to allow sample to fill chamber, reagent flows freely through 0.02 in bypass tube.*

Alternatively, a sample loop bypass injector was constructed to allow sample injection as a discrete plug of reproducible volume into the reagent stream. This injector and flow system is shown in Figure 4 together with the tube dimensions. The injected volume was 30 μl. The position of the stopcock had no effect on the flowing conditions at the detection cell since reagent was able to flow freely through the bypass tube when the main tube was closed. Using this injector, $H_2O_2$ standards were injected directly into the reagent stream and mixing was achieved solely by diffusion of the sample plug.

The third method of injection was by syringe through a septum directly into the reagent stream. This method suffered contamination of the septum port, and gave the lowest precision of the three methods.

The iodine produced by the $H_2O_2$ by reaction 2 was measured in the excess iodide at an applied potential of 200 mV.

*$H_2O_2$ – FIA – platinum wire versus SCE*
Standards of $H_2O_2$ were analysed by measuring the $H_2O_2$ directly by oxidation on platinum in a flowing stream at +05 V against SCE. $H_2O_2$ was prepared in 0.1 M phosphate buffer at pH 8.0, and was injected by syringe into a flowing stream of the same buffer.

*$H_2O_2$ – FIA – platinum or gold wire versus SCE*
Reaction 2 was again the basis for detecting $H_2O_2$. Standards in pH 5.0 acetate buffer were injected with the stopcock injector into a flowing stream of 0.1 M acetate, pH 5.0, 0.1 M KI, and $10^{-3}$M heptamolybdate. Downstream the iodine produced by reaction 2 was reduced at either a platinum or gold wire electrode immersed into the flowing stream. The potential at the electrode was 0.0 V against SCE. The amperometric current was proportional to the initial concentration of $H_2O_2$.

*$H_2O_2$ – CFA – platinum wire versus silver/silver chloride*
$H_2O_2$ standards in pH 5.0 acetate buffer were injected with the stopcock valve into a reagent stream containing 0.1 M acetate, pH 5.0, 0.2 M KI, $10^{-3}$ M heptamolybdate, and $10^{-3}$M KI. The $H_2O_2$ oxidised the iodide to iodine, and the iodine was then reduced at the platinum electrode. The potential of the platinum electrode was +100 mV versus the silver/silver chloride electrode.

### Results and discussion

### Measurement of iodine

$H_2O_2$ is a product of oxidase enzyme catalysed reactions, and indeed, the direct measurement of $H_2O_2$ has been studied and used to determine glucose and other substances [19]. However, at a platinum electrode a potential of at least +0.35 V versus SCE (depending on the pH) is required for the anodic decomposition of the $H_2O_2$.

Aqueous standards of $H_2O_2$ were measured on a platinum wire electrode at pH 5.0 by injecting 20 microlitre volumes into a flowing buffer stream. $H_2O_2$ concentrations between $10^{-3}$ and $10^{-5}$ M were measured satisfactorily. In biological fluids, however, a high background current is encountered when a platinum electrode is poised at 0.5 V and higher potentials. Thus, a method of coupling the $H_2O_2$ to iodide was investigated.

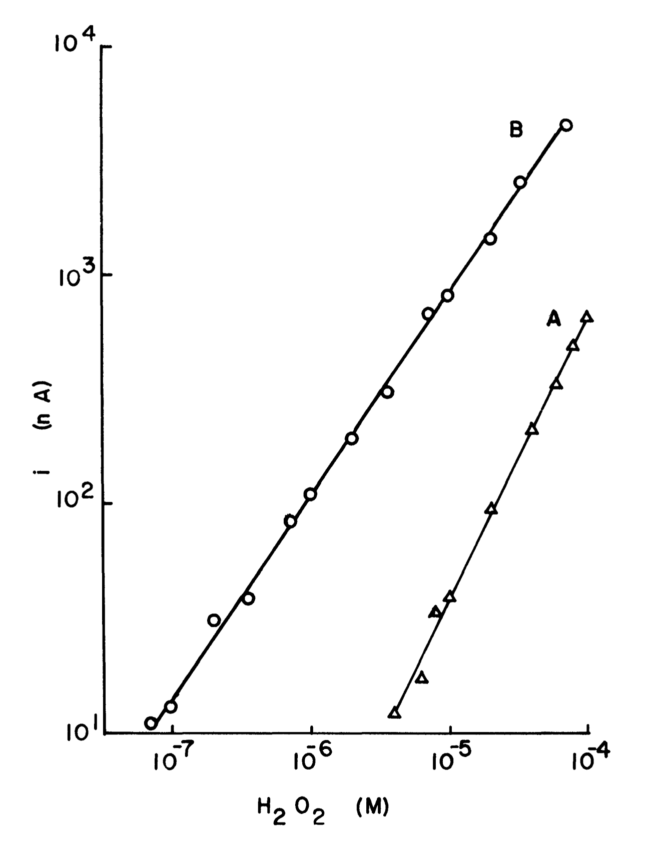

*Figure 5. A: $H_2O_2$ coupled to $I^-$-$I_2$ at pH 7.8 and measured on twin platinum wire electrodes at 0.2 V (CFA). B: $H_2O_2$ coupled to $I^-$-$I_2$ at pH 5.0 and measured on twin platinum wire electrodes at 0.2 V (CFA).*

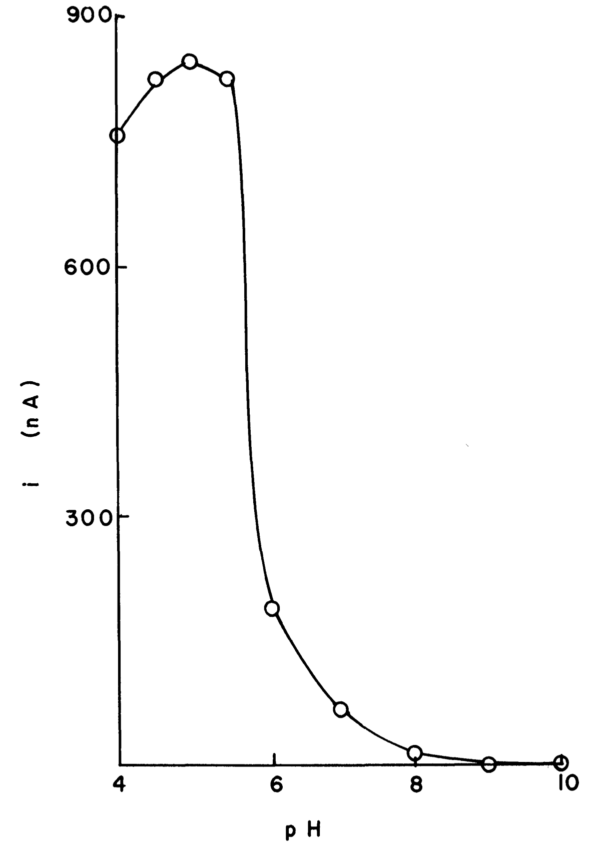

*Figure 6. pH profile for measurement of $H_2O_2$ coupled to $I^-$-$I_2$ and measured at twin platinum wire electrodes at 0.2 V.*

The $I_2$-$I^-$ couple is electroactively reversible, and at twin platinum electrodes poised at a small potential, the $I^-$ is oxidised at the anode while the $I_2$ is reduced at the cathode. A low, 200 mV, potential poised between twin platinum electrodes gives a low background current and a high sensitivity to $I_2$ in the presence of excess $I^-$.

Linearity was obtained for the measurement of $I_2$ in unbuffered aqueous solution over 2 orders of magnitude. There is some upward drift in the current as the standards are successively measured. For instance, the net signals for $10^{-5}$ M $I_2$ in triplicate analysis are 0.18, 0.21, and 0.23 μA, respectively, when the set of $I_2$ standards between $10^{-6}$ and $10^{-4}$ M is run three times in sequence. This problem is discussed in more detail below.

The pH had little effect on the measurement of $I_2$ by our system. However, the blank current was affected, rising smoothly from 25 nA at pH 1 to 50 nA at pH 4 and falling back to 20 nA by pH 7.

**Measurement of $H_2O_2$**

The first series of measurements of $H_2O_2$ was carried out at pH 7.8 because many enzymic reactions have their optimum range around this pH. $H_2O_2$ concentrations down to 4 x $10^{-6}$ M were measured, and linearity was found over 1 – 1/2 orders of magnitude (Figure 5, curve A). The accuracy below $10^{-5}$ M was unsatisfactory using this pH.

Since the reaction 2 consumes $H^+$ it was expected that the reaction would favour a low pH. Indeed the rate of reaction 2 is seen to fall off rapidly above pH 5.5 (Figure 6) and to have its maximum value at pH 5.0. Therefore, another calibration curve for determining $H_2O_2$ was constructed with all standards and the reagent buffered at pH 5.0 (Figure 5, curve B). The detection limit for $H_2O_2$ was lowered by 2 orders of magnitude over the same determination at pH 7.8. The data are linear over the range of concentrations measured (7 x $10^{-8}$ to 7 x $10^{-5}$ M).

**Effect of Molybdate**

Molybdate ion catalyses reaction 2. Figure 7 shows the effect of the $MoO_4^{2-}$ concentration on the rate of the reaction. A 7 x $10^{-5}$ M $H_2O_2$ solution in 0.2 M acetate buffer, pH 5.0, was run through one inlet tube while 0.1 M KI in varying $MoO_4^{2-}$ concentrations was run through the other inlet tube. A heptamolybdate concentration of at least 1 x $10^{-3}$ M (7 x $10^{-3}$ M $MoO_4^{2-}$ ) was required to give maximum sensitivity to $H_2O_2$ measurement. A higher concentration (I x $10^{-2}$ M) of heptamolybdate was found to hydrolyse and precipitate on storing. This can be avoided by preparing the $MoO_4^{2-}$ solutions without addition of the KI.

**Application to ethanol analysis**

An immobilised alcohol oxidase column had been previously used to determine blood ethanol in a flow system by monitoring the oxygen concentration of the effluent from the enzyme column with an oxygen electrode in a flow cell [17]. The same column was used here to analyse aqueous ethanol standards (Figure 8). The biamperometric detection system showed almost an order of magnitude improvement in sensitivity over the former oxygen monitoring system. While the enzyme column used here had much lower activity than previous enzyme columns prepared, (approximately a factor of 5 lower than a freshly prepared column [20]), a

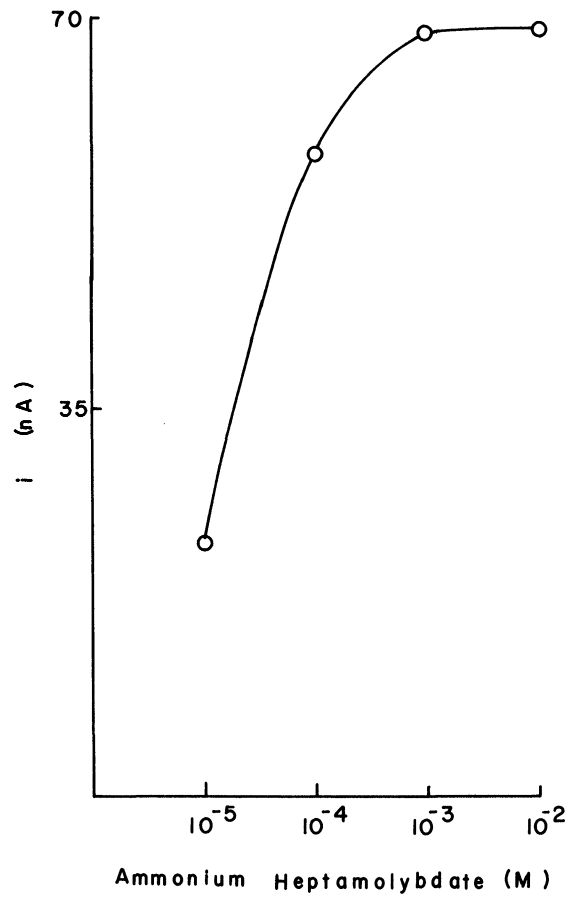

*Figure 7. Effect of Mo(VI) on the reaction between $H_2O_2$ and $I^-$.*

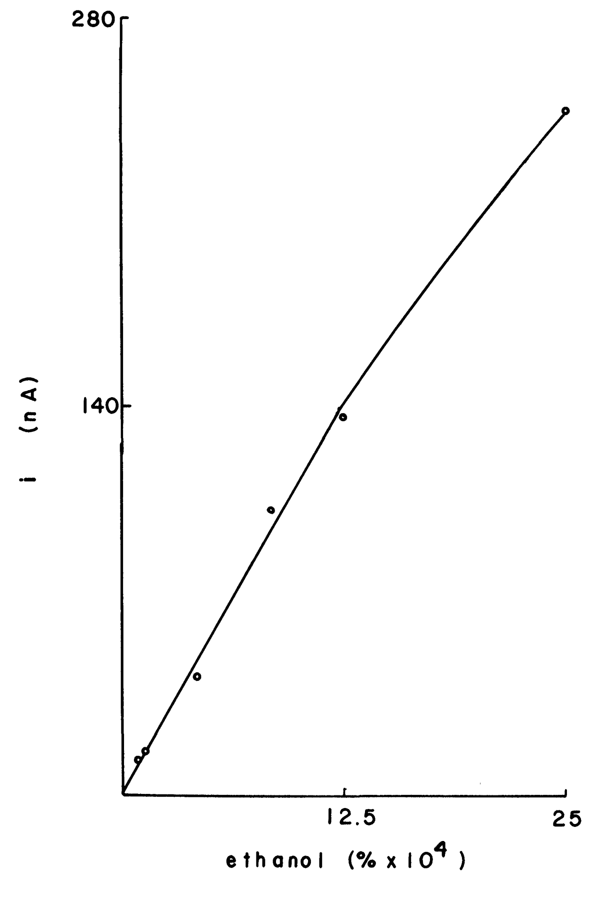

*Figure 8. Continuous flow ethanol determination using flow system shown in Figure 3.*

concentration as low as 0.000083%, with linearity up to 0.0025% ($1.44 \times 10^{-5}$ to $4.34 \times 10^{-4}$M) ethanol was detected.

Since the pH optimum of immobilised alcohol oxidase is 8.2 [21] and the pH optimum for reaction 2 is 5.0, the flow system arrangement in Figure 3 was used. The effluent from the enzyme column was a 0.05 M, pH 8.2 buffer. This was mixed with a 0.2 M, pH 4.7 buffer in approximately a 2 to 1 ratio (determined by the relative flow rates in the two inlet tubes) so that the indicating reaction (reaction 2) occurred at pH 5.0.

**Reproducibility**

Poor reproducibility of the steady state biamperometric signals was obtained in the continuous flow measurement of $I_2$ in the presence of excess $I^-$. As mentioned previously, a $10^{-5}$ M $I_2$ solution gave signals of 0.18, 0.21, and 0.23 $\mu$A when measured at three different times in sequence with other $I_2$ concentrations. On the other hand, the same solution gave net signals of 0.19, 0.19, 0.18, 0.19 and 0.18 $\mu$A (relative standard deviation of 1.6%) when the standard was alternated with the blank. These data suggest iodine poisoning of the electrodes. Iodide has been reported to adsorb onto platinum [18, 22]. With this adsorption as a possible contributor to the irreproducibility of the $I_2$ measurement and $H_2O_2$ determination when coupled to $I_2$-$I^-$, it was decided to employ flow injection analysis of $H_2O_2$ standards was used to determine if the transient presence of $I_2$ would alleviate some of the adsorption problems.

Figure 9 shows the data obtained when $H_2O_2$ standards from $3.5 \times 10^{-7}$ to $3.5 \times 10^{-4}$ M were injected, and the resulting $I_2$ was measured biamperometrically. The average relative deviation of the data is 15%. The data fit a straight line with a correlation coefficient of 0.991.

However, the signals did not vary randomly around the best fit line. There was a definite increasing trend in the signals from a particular $H_2O_2$ concentration when the samples were run singularly from lower to higher concentrations and then the process repeated one or two times. The FIA signals obtained in constructing a calibration curve, similar to that shown in Figure 9, are shown in Figure 10. The problem described above is evident.

A gold plated platinum wire was used as the cathode in an attempt to determine $H_2O_2$ by measuring $I_2$ amperometrically versus an SCE. The same problem as when a platinum cathode was used was encountered.

If iodide adsorption was the true cause of the irreproducibility of the amperometric signals when iodine was reduced at a platinum or gold cathode, a high concentration of chloride (0.2 M) in the reagent buffer containing less iodide ($10^{-3}$ M rather than $10^{-1}$ M) might alleviate the problem.

With a tubular silver electrode at -100mV relative to a platinum wire electrode and a reagent containing the chloride and the iodine a very low (20 nA) background current was obtained. Standards of $H_2O_2$ oxidised iodide to iodine which was then reduced at the platinum electrode. A $6 \times 10^{-6}$ M $H_2O_2$ solution resulted in a biamperometric current 5 nA above the 20 nA background current (Figure 11). The low ($1 \times 10^{-3}$ M) concentration of $I^-$ appears to prohibit the complete reduction of $H_2O_2$ by $I^-$ in the 22 second reaction time allowed. Thus the response of the detection system is non-linear above $2 \times 10^{-5}$ M $H_2O_2$

The sensitivity of this detection system was a factor of 10 worse than that of the twin platinum electrodes but the reproducibility proved to be superior to any of the other $I_2$-$I^-$ detection systems utilised, with an average relative deviation of 6%. However, it must be noted that air bubbles trapped at either electrode causes spurious readings.

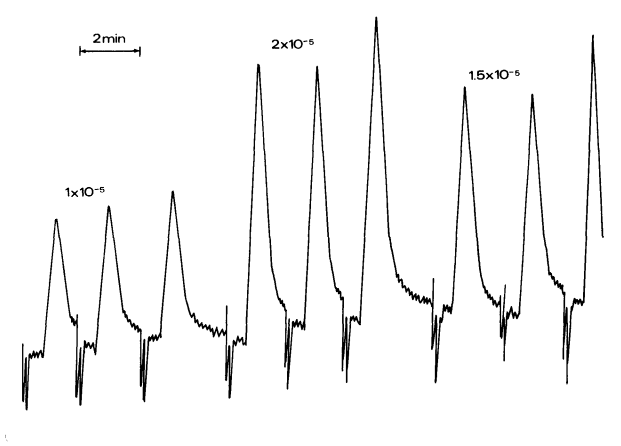

*Figure 10. FIA Signal peaks using stopcock injector with twin platinum wire electrodes.*

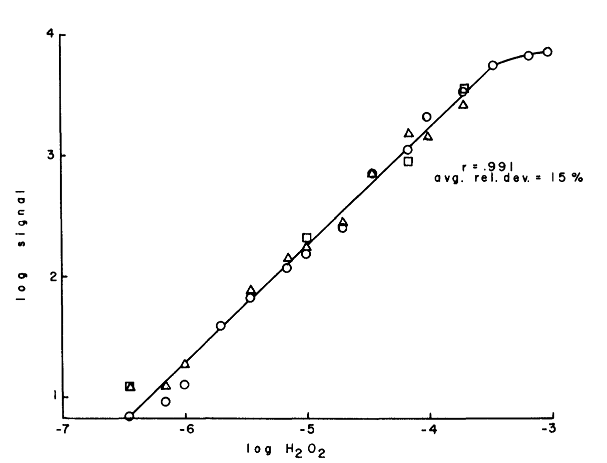

*Figure 9. Peak height for measurement of $H_2O_2$ coupled to $I^-$-$I_2$, measured on twin platinum electrodes at 0.2 V (FIA).*

○ - *first run* △ - *second run* □ - *third run*

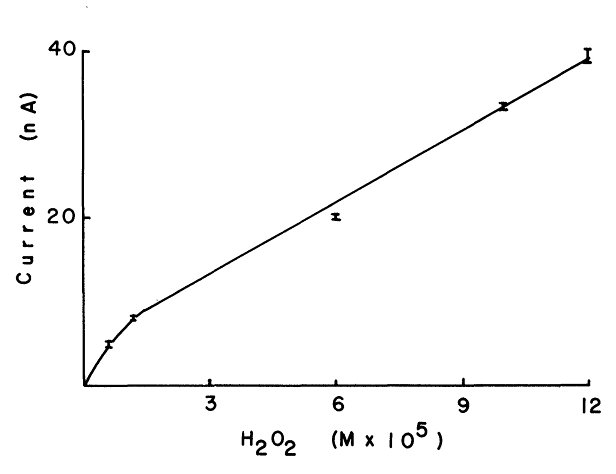

*Figure 11. CFA of $H_2O_2$ coupled to $I^-$ -$I_2$. $I_2$ is reduced at the platinum electrode which is poised at +0.1 V versus Ag/AgCl. Average relative deviation for quadruplicate analyis is 6%.*

### Conclusion

It has been demonstrated that the electrochemical detection of iodine in the presence of excess iodide can be reliable and sensitive, when the appropriate solution environment is used with the appropriate working and counter electrodes. While certain electrode systems have a very low detection limit for iodine, they can suffer poor reproducibility due to absorption onto or possibly oxidation of the electrode surfaces. One electrode system has been found to offer good sensitivity and repeatability.

$H_2O_2$, a product of oxidase enzyme reactions, can be sensitively coupled to the reversible $I_2$-$I^-$ redox pair, offering a method for determining certain substrates of interest.

Sample volumes around 25 $\mu$l can be analysed at sampling rates typically of about 30 per hour, but as high as 80 per hour, by flow injection analysis.

REFERENCES

[1] Schwartz M. K., *Analytical Chemistry*, 1973, **45**, 739A.
[2] Betteridge D., *Analytical Chemistry*, 1978, **50**, 832A.
[3] Snyder L., Levin J., Stoy R. and Conetts A., *Analytical Chemistry*, 1976, **48**, 942A.
[4] Bergmeyer H.U. and Hagen A., *Analytical Chemistry*, 1972, **261**, 333.
[5] Ruzicka J. and Hansen E. H., *Analytica Chimica Acta*, 1976, 87, 353.
[6] Stewart K. K., Beecher G. R. and Hare P. E., *Analytical Biochemistry*, 1976, **70**, 167.
[7] Basson W. D. and Van Staden J. F., *Analyst*, 1978, **103**, 296.
[8] Nikolelis D. P. and Mottola H. A., *Analytical Chemistry*, 1978, **50**, 1665.
[9] Wolff C. H. and Mottola H. A., *Analytical Chemistry*, 1978, **50**, 95.
[10] Bailley P. L., *Analytical Chemistry*, 1978, **50**, 698A.
[11] Stulik K. and Vaclav V., *Journal of Electroanalytical Chemistry*, 1978, **70**, 253.
[12] Attiyat A. S. and Christian G. D., *Chemical, Biomedical and Environmental Instrumentation*, 1979, 9, 261.
[13] Attiyat A. S. and Christian G. D., *Analytica Chimica Acta*, 1979, **106**, 225.
[14] Attiyat, A. S., PhD Thesis, University of Washington, 1979.
[15] Pantel S. and Weisz H., *Analytica Chimica Acta*, 1977, **89**, 47.
[16] Pardue H. L., *Analytical Chemistry*, 1963, **35**, 1240.
[17] Gulberg E. L. and Christian G. D., *Chemical, Biomedical and Environmental Instrumentation*, 1979, 9, 277.
[18] Weetall H. H. in 'Methods in Enzymology' Ed. K. Mosbach, 1976, Vol. XLIV, Academic Press, London.
[19] Guilbault G. G. and Lubrano J. J., *Analytica Chimica Acta*, 1973, **64**, 439.
[20] Gulberg E. L. and Christian G. D., *Analytica Chimica Acta*, in press.
[21] Kissinger P. T., *Analytical Chemistry*, 1977, **49**, 488A.
[22] Lingane J. J., 'Electroanalytical Chemistry', 1958, Interscience Publishers, New York.

### Interfacing a titrator to a microcomputer for incremental or continuous modes of operation

**M. Danczigerand H. V. Malmstadt***

*School of Chemical Sciences, University of Illinois, Urbana, Illinois 61801, USA*

There are several microcomputer-controlled titrators now on the market. Some of these have been programmed to utilise many methods of endpoint detection, including the incremental titrant delivery and calculation techniques. However, these incremental methods are not readily implemented on older titrators. It is the purpose in this paper to describe some rather simple interface designs and automation methodology that enable a few conventional titration modules to be interconnected with a microcomputer so as to provide an 'intelligent' and versatile automated titrator. This system is then used to provide some comparisons of the various incremental titrant delivery and calculation modes. It can also be used in the continuous delivery mode to a preset [1] or derivative [2,3] endpoint. Several concepts of a microcomputer-controlled titrator and selection of an endpoint calculation technique are illustrated.

Although the theoretical aspects of the Kolthoff [4], Fortuin [5], Wolf [6], Keller-Richter [7], and Bartscher [8] incremental methods have been discussed, there is little information in the literature on practical comparisons. The automated titrator described here has enabled hundreds of unbiased titration results to be printed out rapidly for the various incremental techniques. Results are presented and discussed for a weak acid-strong base and a precipitation titration. The incremental methods are compared on the basis of the experimental results obtained.

*Correspondence to this author

### Instrumentation

A block diagram of the automated titrator is shown in Figure 1. An ADD-8080 microcomputer [9] provides the "intelligence" for the titrator. It is based on the 8080A microprocessor (Intel Corp., Santa Clara, Calif. 95051, USA). The microcomputer has 10K bytes of programmable read only memory (PROM) which contain a BASIC interpreter (a modification of BASIC/5, Processor Technology Corp., Emeryville, Calif. 94608, USA), a monitor program to facilitate machine language programming, and several utility programs. Also present are 15K bytes of read-write memory (RWM) which are used to store BASIC user programs in machine language and data. An arithmetic processing unit (AM9511 APU, Advanced Micro Devices, Inc., Sunnyvale, Calif. 94086, USA), is available to perform calculations that would be too time-consuming or cumbersome if done on the microprocessor. A conventional teletypewriter provides interaction with the operator.

